# Silencing TAK1 reduces MAPKs-MMP2/9 expression to reduce inflammation-driven neurohistological disruption post spinal cord injury

**DOI:** 10.1038/s41420-021-00481-5

**Published:** 2021-05-08

**Authors:** Shuai Jiang, Yandan Wu, Shunjie Wu, Suhui Ye, Renyi Kong, Jie Chang, Mingjie Xia, Junping Bao, Xin Peng, Xin Hong, Zhanyang Qian, Haijun Li

**Affiliations:** 1 Spine Center, Zhongda Hospital of Southeast University, Nanjing, China; 2 Department of Microbiology and Immunology, Medical School, Southeast University, Nanjing, China; 3 Department of Orthopedics, First Affiliated Hospital of Nanjing Medical University, Nanjing, China; 4 Department of Orthopedics, Nanjing First Hospital, Nanjing Medical University, Nanjing, China; 5 Department of Orthopedics, Taizhou Clinical Medical School of Nanjing Medical University, Taizhou People’s Hospital, Taizhou, China

**Keywords:** Post-traumatic stress disorder, Acute inflammation

## Abstract

Microglia activation post traumatic spinal cord injury (SCI) provokes accumulation of inflammatory metabolites, leading to increasing neurological disruption. Our previous studies demonstrated that blocking MAPKs pathway mitigated microglia inflammatory activation and prevented cords from neuroinflammation-induced secondary injury. Transforming growth factor-β-activated kinase 1 (TAK1) is an upstream gate regulating activation of MAPKs signaling. To validate the therapeutic effect of TAK1 inhibition in neuroinflammation post SCI, in the current study, cultures of microglia BV2 line was undergone lipopolysaccharide (LPS) stimulation in the presence of TAK1 inhibitor 5Z-7-Oxozeaenol (ZO), LPS, or control. LPS triggered inflammatory level, cell migration, and matrix metalloproteinase (MMP) 2/9 production, which was reduced in ZO-treated cultures. TAK1 inhibition by ZO also decreased activation of MAPKs pathway, indicating that ZO-mediated alleviation of neuroinflammation is likely modulated via TAK1/MAPKs axis. In vivo, neuroinflammatory level and tissue destruction were assessed in adult male mice that were undergone SCI by mechanical trauma, and treated with ZO by intraperitoneal injection. Compared with SCI mice, ZO-treated mice exhibited less microglia pro-inflammatory activation and accumulation adjacent to injured core linked to reduced MMP2/9 expression, leading to minor tissue damage and better locomotor recovery. To sum up, the obtained data proved that in the early phase post SCI, TAK1 inhibition impedes microglia biological activities including activation, enzymatic synthesis, and migration via downregulation of MAPKs pathway, and the effects may be accurately characterized as potent anti-inflammation.

## Introduction

Microglia, as a resident immune cell in neural tissue, plays a vital role in inflammatory and immune responses post neurological injury^1,2^. Following a traumatic spinal cord injury (SCI) inducing primary mechanical contusion of neural tissue, resting microglia rapidly activate and rally to the lesion with overproduction of pro-inflammatory factors to stimulate neuroinflammation^3–5^. Next, activated microglia works together with infiltrating leukocytes from the peripheral circulation to release an increased quantity of pro-inflammatory mediators and reactive oxygen species (ROS) while allowing higher infiltration of peripheral leukocytes to exacerbating secondary tissue injury surrounding the primary lesion^6^. The current clinical treatment for SCI is still administrated large dose of methylprednisolone, but increasing researchers have suggested that the routine ictus treatment not only did not significantly inhibit the acute neuroinflammation, but also the side effects of treatment had a great burden on the patient’s body^7,8^. How to effectively increase control over acute neuroinflammation post traumatic nerve injury to improve tissue repair is a contentious unresolved issue.

Transforming growth factor-β-activated kinase (TAK1) is originally defined as a mitogen-activated protein kinase kinase kinase (MAP3K) activated by TGF-β^9^. Since this crucial report, TAK1 subsequently has been found to regulate various signaling pathways, including inflammation, apoptosis, and oxidative stress^10–12^. In inflammatory response, TAK1 activation by microbial lipopolysaccharide (LPS), TNF-α, and IL-1 phosphorylates the complex of IKK, which further degrades IκB, leading to activation of NF-κB^13–15^. Phosphorylation of TAK1 also activates MAPKKs, resulting in activation of MAPKs signaling pathways^16^. ZO is a classic TAK1 irreversible inhibitor through forming a covalent complex with TAK1^17^. It was reported that ZO attenuates the development of inflammation and tissue damage in a number of experimental disease models^17–19^. Given the previous presence, we hypothesize that inhibition of TAK1 by ZO serves as an effective targeted therapy against microglia-induced neuroinflammation and oxidative stress post SCI.

In the current study, we verified a previously unidentified effect for TAK1 inhibition on microglia-induced neuroinflammation and tissue degradation after SCI. Also, a potent target was found for control over neuroinflammation post SCI through the MAPKs signaling pathways. Meaningfully, our findings systematically display that suppression of microglia-driven neuroinflammation exerts important functions on protection against secondary injury and promotion for further pathological healing during the progression of SCI.

## Results

### High phosphorylation of TAK1 and p38 in vivo and in vitro during neuroinflammation

**Fig. 1:**
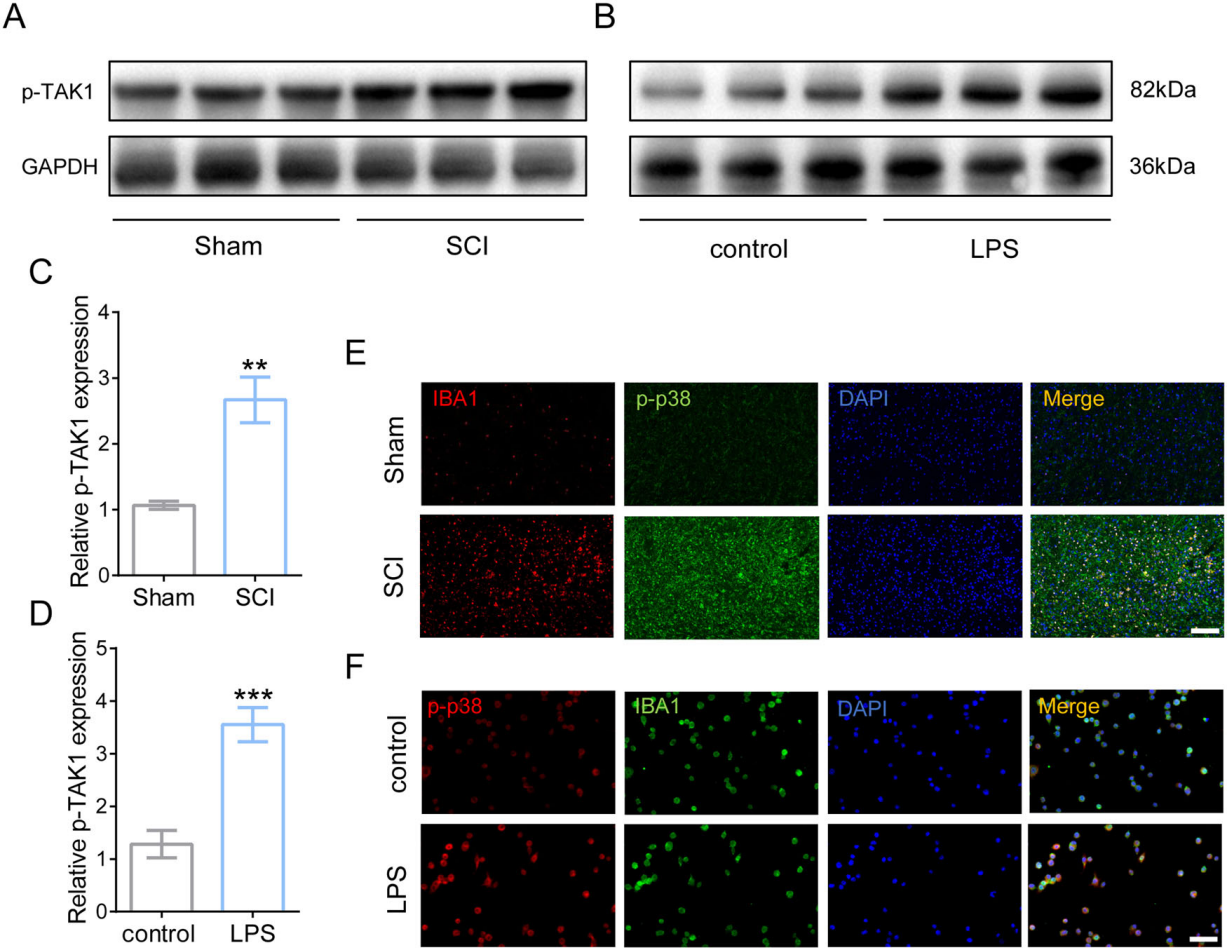
The expression of phosphorylation of TAK1 and p38 in vivo and in vitro increased during neuroinflammation response. Western blotting of phosphorylated TAK1 (p-TAK1) in vivo SCI model **A** and in vitro LPS-treated microglia (**B**). **C** Quantification of p-TAK1 in vivo. ^∗∗^*P* < 0.01 vs. sham group; *n* = 6 in each group. Data were collected in three independent experiments. **D** Quantification of p-TAK1 in vitro. ^∗∗∗^*P* < 0.001 versus control. Data are presented as mean ± SD, *n* = 6 for each bar. **E** Representative IF images of phosphorylated p38(p-p38) in the injured cord; Bar scale = 100 μm. **F** Representative IF images of p-p38 were in LPS-mediated microglial neuroinflammation; Bar scale = 50 μm.

To elucidate whether TAK1 activation involves in neuroinflammatory response, the expression of phosphorylated TAK1 (p-TAK1) was measured in vivo SCI models and in vitro LPS-treated microglia by WB after 24 h. It was found that phosphorylated TAK1 increased significantly following SCI modeling in mice and LPS treatment in microglia (Fig. 1A–D). Furthermore, the activation of a downstream signaling conductor p38 was visualized using IF, showing that p38 was markedly activated in injured cord, especially in ionized calcium-binding adaptor protein 1 (IBA1) labeled microglia (Fig. 1E). In vitro, high expression of phosphorylated p38 (p-p38) was also observed in LPS-mediated microglial neuroinflammation (Fig. 1F). The results showed that extensive activations of TAK1 and p38 were witnessed in neuroinflammatory microglia both in vivo and in vitro.

### Inhibition of TAK1 blocks pro-inflammatory transition of microglia by reducing MAPKs pathway

**Fig. 2:**
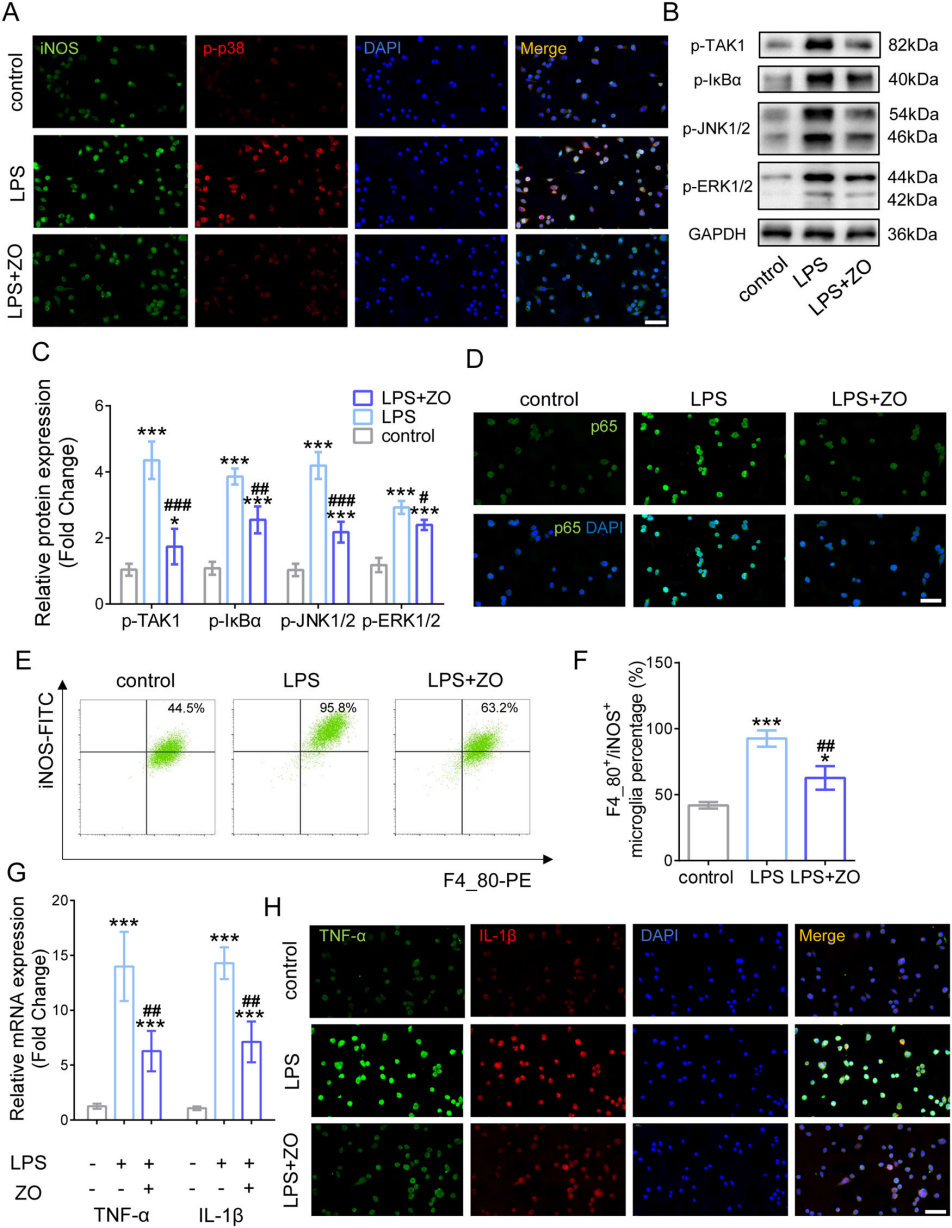
Blocking TAK1 attenuates microglial inflammation by reducing MAPKs pathway. **A** Representative IF images of inducible nitric oxide synthase (iNOS) and p-p38; Bar scale = 50 μm. **B** Western blotting of phosphorylation of TAK1, IκBα (p-IκBα), JNK1/2 (p-JNK1/2), and ERK1/2 (p- ERK1/2) after TAK1 inhibition. **C** Quantification of p-TAK1, p-IκBα, p-JNK1/2, and p- ERK1/2. ^∗^*P* < 0.05, ^∗∗∗^*P* < 0.001 versus control, # *P* < 0.05, ## *P* < 0.01, ### *P* < 0.001 versus L*P*S group. **D** Representative IF images of p65 in LPS-treated microglia after TAK1 inhibition; Bar scale = 50 μm. **E** FCA results of F4_80-PE positive and iNOS-FITC positive cells. **F** Quantification of iNOS-FITC positive microglia ratio after ZO administration. **G** Quantification of tumor necrosis factor (TNF)-α and interleukin (IL)−1β. **H** Representative IF images of TNF-α and IL-1β under TAK1 inhibition after LPS treatment; Bar scale = 50 μm.

After LPS treatment for 24 h, inducible nitric oxide synthase (iNOS), a pro-inflammatory marker of microglia, and p-p38 were examined by IF. Interestingly, we found that neuroinflammatory activation increased iNOS and p-p38 expressions in microglia yet inhibition of TAK1 by ZO treatment reduced iNOS and p-p38 protein levels in activated cells (Fig. 2A). Moreover, WB showed that activation of other inflammation-related pathways, including IκBα, JNK1/2, and ERK1/2, were decreased after TAK1 inhibition during neuroinflammatory response as well (Fig. 2B, C). Importantly, reduced expression of p65 was presented in LPS-treated microglia after ZO employment (Fig. 2D). FCA results exhibited a dramatic increase in F4_80-PE positive and iNOS-FITC positive cells following LPS stimulation but a remarkable inhibition of iNOS-FITC positive microglia ratio by ZO administration (Fig. 2E, F), indicating that decreased TAK1 activation inhibited inflammatory phenotype of microglia. It was shown that the RNA expressions of classic inflammatory cytokines, such as tumor necrosis factor (TNF)-α and interleukin (IL)−1β, prominently decreased in TAK1-inhibited microglia after LPS utilization for 12 h (Fig. 2G). Consistently, IF displayed that the protein expressions of TNF-α and IL-1β were reduced in activated microglia under TAK1 suppression after LPS treatment for 24 h (Fig. 2H). Collectively, blocking TAK1 attenuates microglial inflammation via inhibiting MAPKs-related signaling axis.

### Disruption of TAK1 reduces microglia migration and matrix metalloproteinases (MMPs) production

**Fig. 3:**
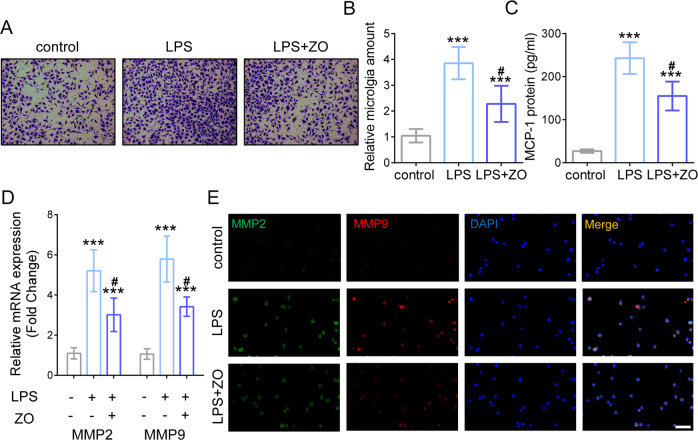
Disruption of TAK1 inhibits microglia migration and MMPs production. **A** Trans-well assay with LPS and ZO administration treated as indicated. **B** The amount of microglia under LPS and TAK1 inhibition treatment. ^∗∗∗^*P* < 0.001 versus control, # *P* < 0.05 versus LPS group. **C** Quantification of Monocyte chemoattractant protein-1 (MCP-1). ^∗∗∗^*P* < 0.001 versus control, # *P* < 0.05 versus LPS group. **D** The RNA levels of MMP2 and MMP9 after LPS inducement and ZO employment. ^∗∗∗^*P* < 0.001 versus control, # *P* < 0.05 versus L*P*S group. **E** Representative IF images of MMP2 and MMP9; Bar scale = 50 μm.

To verify whether restraining TAK1 affects inflammation-driven microglial migration, trans-well assay was carried out to evaluate the capacity of microglia. As shown in Fig. 3A, LPS-mediated inflammation provoked more migrated microglia, while TAK1 inhibition by ZO significantly decreased the amount of microglia compared with the LPS group (Fig. 3B). Besides, the representative chemokine monocyte chemoattractant protein-1 (MCP-1) was found to be increased signally after LPS treatment. However, disrupting TAK1 activation reduced MCP-1 expression (Fig. 3C). MMPs generation was measured at the levels of both transcription and translation. Results showed that the RNA and protein levels of MMP2 and MMP9 elevated by LPS inducement, whereas ZO employment inhibited their expressions during microglial inflammation (Fig. 3D, E). Taken together, TAK1 blocking suppresses microglia migration and MMPs production during inflammatory response.

### TAK1 inhibition alleviates microglia-induced neuroinflammation and MMP-induced tissue destruction

**Fig. 4:**
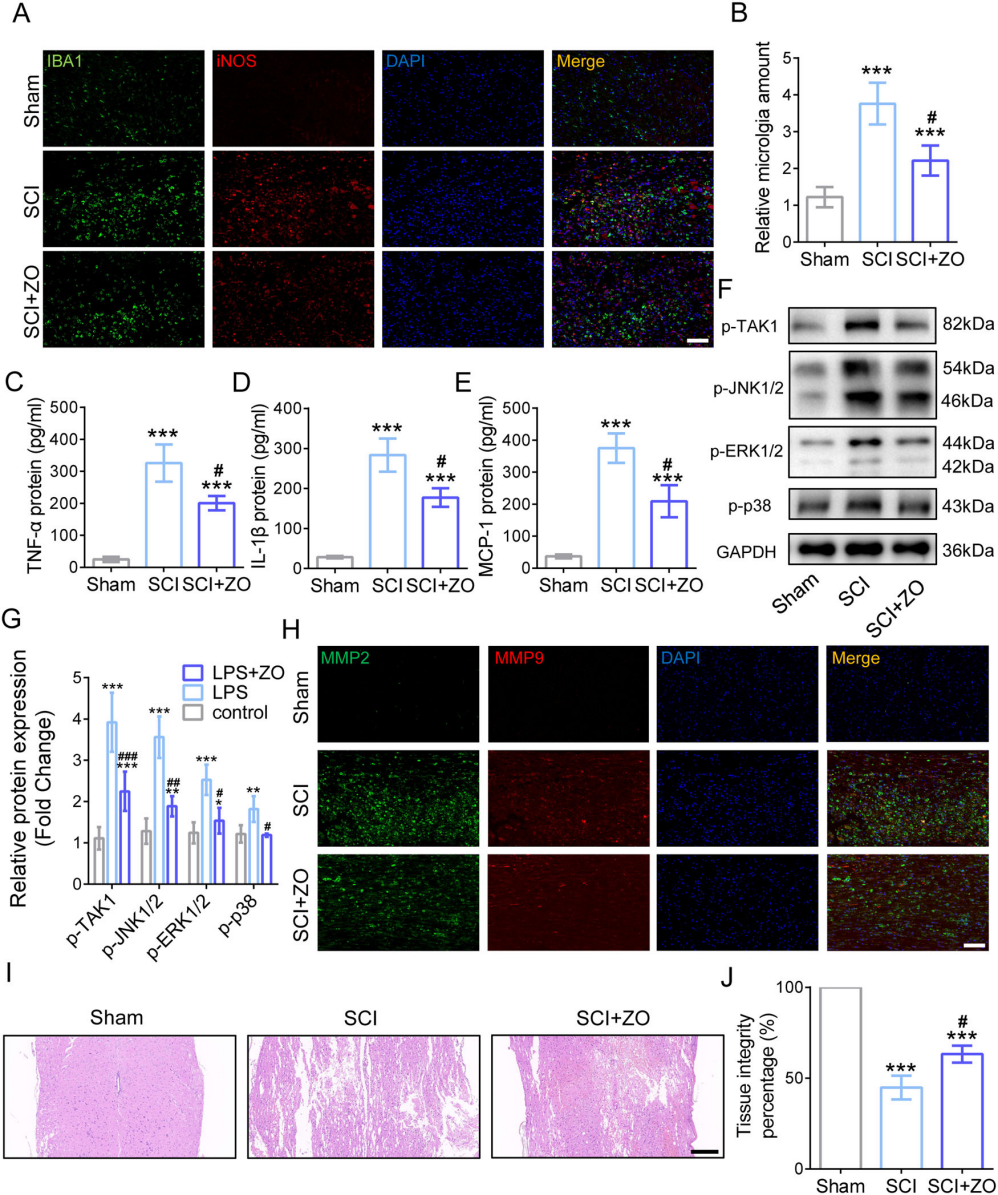
TAK1 inhibition ameliorates neuroinflammation and tissue destruction caused by microglia and MMPs generation. **A** Representative IF images of IBA1 and iNOS after LPS inducement and ZO administration; Bar scale = 100 μm. **B** The amount of microglia under LPS and TAK1 inhibition treatment. ^∗∗∗^*P* < 0.001 versus Sham group, # *P* < 0.05 versus SCI group. The protein levels of TNF-α, IL-1β, and MCP-1. ^∗∗∗^*P* < 0.001 vs. Sham group, # *P* < 0.05 vs. SCI group **C**–**E**. **F** Western blotting of p-JNK1/2, p-ERK1/2 and p-p38. **G** Quantification of p-JNK1/2, p-ERK1/2, and p-p38. ^∗^*P* < 0.05, ^∗∗∗^*P* < 0.001 versus Sham group, # *P* < 0.05, ## *P* < 0.01, ### *P* < 0.001 versus SCI group. **H** Representative IF images of the expressions of MMP2 and MM*P*9 in injured cords; Bar scale = 100 μm. **I** Representative HE staining photos of spinal cord in Sham, SCI, SCI + ZO group at 3dpi; Bar scale = 200 μm. **J** Quantification of tissue integrity in the spinal cord.

At 3 days post SCI (dpi), the inflammatory phenotype and the amount of microglia (longitudinal sections centered around the injured core 1 mm) was measured using IF, showing that IBA1-positive area increased after SCI while TAK1 inhibition markedly reduced the accumulation of microglia (Fig. 4A, B). More notably, the cells co-staining by IBA1 and iNOS exhibited a decrease in SCI mice under TAK1 inhibition, implying that TAK1 suppression reduced inflammatory microglia activation post SCI (Fig. 4A). Moreover, the inflammatory cytokines and chemokine, including TNF-α, IL-1β, and MCP-1, were examined using ELISA, exhibiting that blocking TAK1by ZO administration decreased their protein levels in SCI mice (Fig. 4C–E). WB analysis showed an increase in MAPKs pathways, including p-JNK1/2, p-ERK1/2, and p-p38 post SCI, whereas decreased trends of them were observed in injured cords after TAK1 invalidation (Fig. 4F, G). The expressions of MMP2 and MMP9 in injured cords were detected using IF, showing that SCI causes the excess generation of MMP2/9 yet TAK1 inhibition post SCI alleviated MMP2 and MMP9 expressions in SCI mice (Fig. 4H). Histologically, HE staining exhibited more severe destruction of cord tissue in SCI mice compared with those in SCI + ZO group as well (Fig. 4I, J). These all results demonstrated that TAK1 inactivation prevents injured cords from tissue destruction caused by microglia-induced neuroinflammation and MMPs generation.

### TAK1 inhibition reduces glial forming and improves neurological recovery in SCI mice

**Fig. 5:**
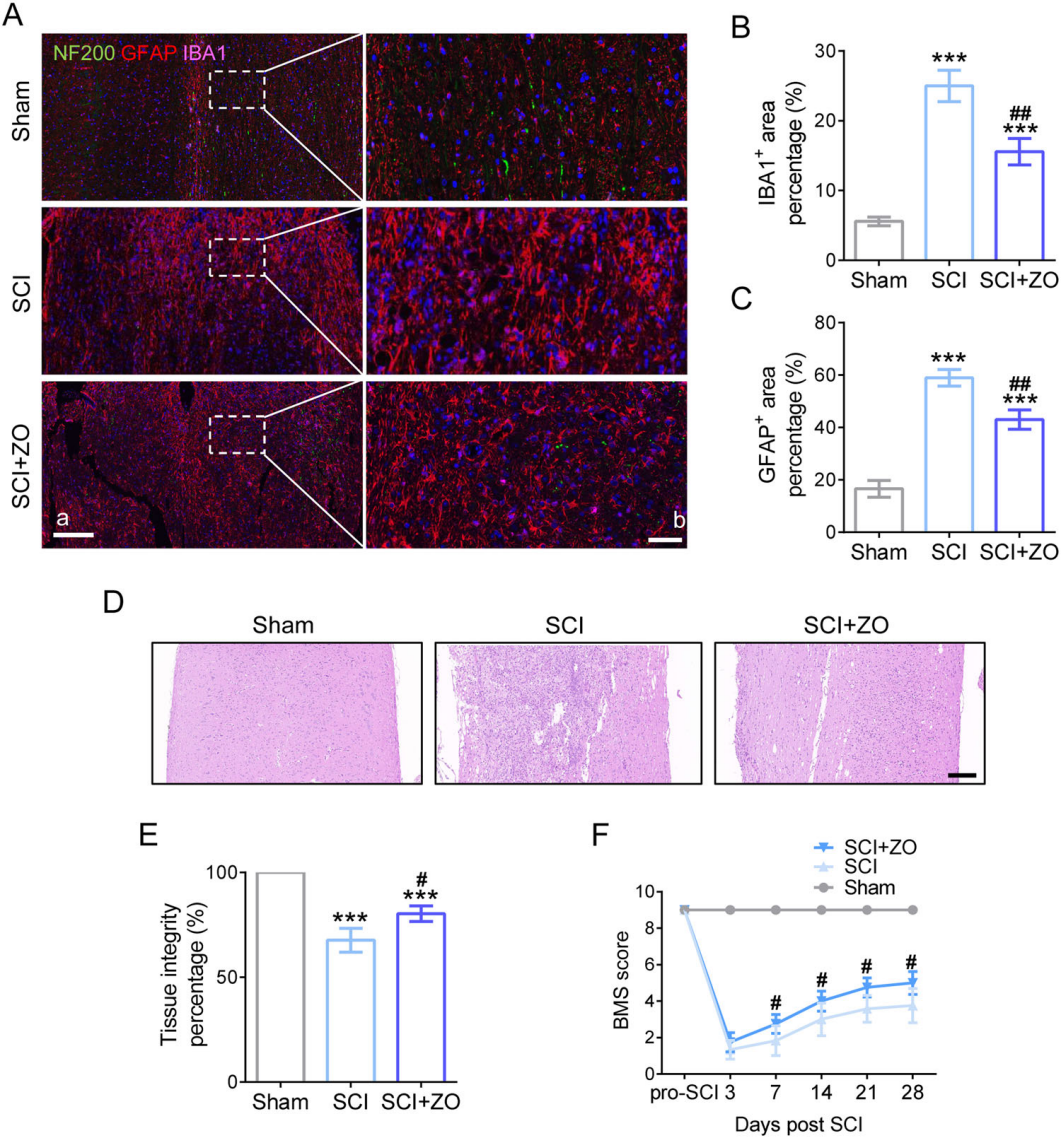
TAK1 inhibition exerts a beneficial effect on histological structure and neurological recovery in SCI mice. **A** Trinary immunofluorescence shows the distribution of NF200 (green), GFAP (red), and IBA1 (pink) in the lesion site of the spinal cord at 28 dpi; a, Scale bar = 200 μm; b, Scale bar = 50 μm. **B** Quantification of the IBA1-positive area in the spinal cord at 28 days post-SCI. ^∗∗∗^*P* < 0.001 versus Sham group, ## *P* < 0.01versus SCI group; *n* = 6 in each group. **C** Quantification of the GFAP-positive area in the spinal cord at 28 days post-SCI. **D** Representative HE staining photos of the spinal cord in Sham, SCI, SCI + ZO group at 28 dpi; Bar scale = 200 μm. **E** Quantification of tissue integrity in the spinal cord. **F** The BMS score post SCI, # *P* < 0.05 versus SCI group.

At 28 dpi, the glial scar components (IBA1 and GFAP labeled) and axons (NF200 labeled) were presented using tricolor IF staining. As shown in Fig. 5Ab, minor areas of microglia (pink) and astrocytes (red) and some axons (green) were witnessed in spinal cord tissue (longitudinal sections centered around the injured core 2 mm in Fig. 5Aa). In SCI group, a mass of glial scar composed of microglia and astrocytes occupied most of the spinal cord area with almost nonexistent axons. However, it was found with less glial scar area and some regenerated axons in SCI mice treated with TAK1 inhibition (Fig. 5A–C). From a perspective on histology, a less glial infiltration around the injured cord and better tissue integrity was observed in TAK1 inhibitor-treated spinal cord (Fig. 5D, E). Functionally, based on the evaluation in accordance with BMS score, the locomotor recovery of the hindlimb in SCI mice was significantly improved by TAK1 inhibition, which appeared at 7 dpi and then peaked at 28 dpi (Fig. 5E). To sum up, blocking TAK1 activation ameliorates histological structure and benefits to neurological recovery post SCI.

## Discussion

Mechanical contusion in the spinal cord causes a string of pathophysiological cascades launching secondary SCI. Thereinto, neuroinflammation voluntarily activates in response to primary injury^20,21^. However, excess inflammatory impact from microglia in situ triggers extensive destruction of histology and deterioration of motor function in the context of SCI^22–24^. Given its crucial clinical implications, it is desired for seeking a promising target to control over post-SCI neuroinflammation. Herein, our research concentrated on the attenuation of microglia-derived neuroinflammation and the preservation of neurological integrity by pharmacological inhibition of TAK1.

TAK1, encoded from the MAP3K7 gene, is located at the cellular hub where TNF-α, TGF-β, and Wnt axis converge^25,26^. To date, TAK1 has been certified as an essential interchanger of inflammatory response in various disease models. The reduction of TAK1 prevents lung inflammation in pneumoconiosis and reduces monosodium urate-induced inflammation as well as obesity-linked inflammation^18,27,28^; however, its biological function was little reported in neurological lesion especially in SCI. It is universally known that TAK1 is activated by a component composed of TAK1-binding protein 2 (TAB2) and RIPK1 polyubiquitin chain. Then activated TAK1 further phosphorylates IKKα/ IKKβ/IKKγ complex, which degrades IκB protein, leading to NF-κB activation^29,30^. On the other hand, TAK1 activation promotes phosphorylation of MAPKKs, resulting in activation of MAPKs pathway, including JNK, ERK, and p38^31^. NF-κB and MAPKs jointly incur downstream generation of inflammatory substances and degrading enzymes such as MMPs^32,33^. Our findings showed that p-TAK1 level was increased, along with p38 activation, in injured spinal cords and cultured BV2 microglia after modeling for 24 h. Interestingly, ZO selectively inhibited phosphorylation of TAK1 and p38 in microglia, and reduced iNOS expression as well as the amount of pro-inflammatory cells. These results suggested a cellular mechanism that TAK1 inhibition partly blocked MAPKs pathway to decrease microglial response to inflammatory mediators.

MAPKs pathway represents an indispensable link in inflammatory signaling so that targeted regulation for MAPKs pathway mitigates advance of multiple diseases under a circumstance of inflammation, including cancer, autoimmune disease, and infectious disease^34–36^. Our previous studies logically reported pharmacological or genetic interventions towards MAPKs pathway corrected skeletal muscle fibrosis and SCI as well^37,38^. We here found that TAK1, as an upstream target of MAPKs pathway, plays a potent regulation for microglia-induced neuroinflammation post SCI. As shown in our data, activation of not only p38 but also JNK and ERK was significantly inhibited after TAK1 inactivation in vitro and in vivo. Thus, reduced inflammatory cytokines like TNF-α and IL-1β were witnessed in microglia during neuroinflammation. It is non-negligible that microglia migrate from the distal to the proximal of injured core in that the process concurrently generates redundant inflammatory cytokines and chemokines for summon of glial cells and peripheric cells, such as astrocytes, macrophages, and neutrophils, instead of functional neural cells. In parallel studies worked in vitro, microglia migration aggravated when TAK1 was activated but attenuated as TAK1 inhibited by ZO. More notably, the employment of ZO to pharmacologically interfere with TAK1 activation concurrently mitigated MCP-1 expression.

MMPs are a type of enzymes that dissolve extracellular matrix under inflammation, tumor, and other pathological conditions^39,40^. In the early phase of inflammation, outbreak of inflammatory cytokines promotes MMPs expression, which afterwards destructs basement-membrane for infiltration of inflammatory cells into deeper or larger tissue. Undoubtedly, microglia produce massive MMPs to exacerbate neural tissue destruction when activation in inflammatory state^41^. MMP2/9 have been widely identified as a group of agitators in tumor metastasis by promoting matrix breakdown to accelerate cell migration when pro-inflammatory mediators stimulated^42,43^. Activation of NF-κB and MAPKs pathways including JNK, ERK, and p38 upregulates MMP2/9 expression; however, selectively inhibiting one of them leads to a reduction of MMP2/9 production^44,45^. Evidence showed that the microglial inflammatory state facilitates roles of matrix-dissolving enzymes for migration^46^. Naturally, through inhibiting neuroinflammation by TAK1 blocking in microglia, we observed a disruptive MMP2/9 production after SCI. The present study associated TAK1/MAPKs axis with microglial biological activity in neuroinflammation, in which TAK1 inhibition alleviated microglia migration along both lines, reducing MCP-1 level and blocking MMP2/9 expression. Also, selectively inhibition of TAK1 prevented severe histological loss at acute inflammatory period post SCI and possibly achieved better recovery of cord histology and function in ZO-treated mice than the untreated mice.

Taken together, TAK1inhibition exhibited a valid suppression of MAPKs activation and control of inflammatory arousal in microglia. However, indiscriminative pharmacological inhibition of TAK1 cannot thoroughly testify the specific superiority and inferiority in other neural cells. In addition, TAK1 is reported to involve in the development of apoptosis, oxidative stress, and autophagy^10,47^, comprehensive and in-depth studies thereby need to be carried out for systematic evaluation of TAK1 in SCI treatment.

## Materials and methods

### Reagent and antibodies

ZO and LPS were obtained from MedChemExpress (Shanghai, China) and the culture medium was purchased from Servicebio (Wuhan, China). Fetal bovine serum (FBS) and Mouse ELISA Kits were purchased from Invitrogen (Carlsbad, CA, USA). Hematoxylin-eosin (HE) staining kit (G1005) and 4% paraformaldehyde (PFA) were purchased from Servicebio. Dimethylsulfoxide (DMSO) was purchased from Solarbio (Beijing, China). TRIzol was purchased from YiFeiXue Biotech (Nanjing, China).

Anti-p-TAK1 (#9339; 1:1000 for western blot (WB)), Anti-p-p38 (#4511; 1:1000 for WB; immunofluorescence (IF) 1:500), Anti-p-IκBα (#2859; 1:1000 for WB), Anti-p-ERK1/2 (#4370; 1:1000 for WB), Anti-JNK1/2 (#4695; 1:1000 for WB), Anti-p65 (#8242; 1:600 for IF), Anti-GFAP (#3670; 1:600 for IF) were from Cell Signaling Technology (Danvers, MA, USA). Anti-IBA1 (#ab178847; 1:500 for IF), Anti-iNOS (#ab15323; 1:100 for IF), Anti-neurofilament heavy polypeptide (NF-200; #ab207176; 1:200 for IF), Anti-MMP2 (ab92536; 1:250 for IF), Anti-MMP9 (ab38898; 1:300 for IF) were from Abcam (Cambridge, MA, USA). Anti-F4_80-PE (565410) and iNOS-FITC (610330) were from BD Biosciences (Franklin Lakes, NJ, USA). Alexa Fluor 488-, 555- and 647-conjuated secondary antibodies (1:500 for IF) was from Jackson (Philadelphia, PA, USA). Anti-GAPDH (HRP-60004; 1:10000 for WB), HRP-conjugated secondary antibodies (1:10000 for WB) were from Proteintech (Chicago, IL, USA).

### Cell culture and treatment

A BV2 microglia line was obtained from the Institute of Cell Research, Chinese Academy of Medical Sciences Medical Science and cultured with Dulbecco’s modified Eagle medium (DMEM) containing 10% FBS in a cell incubator at 37 °C with 5% CO_2_. Microglia were carried out starvation after confluence reached 80%, followed by pretreatment with ZO (1 μM, dissolved in 0.1% DMSO) for 2 h. Then LPS (100 ng/mL) was employed to stimulate microglia activation for a required time.

### SCI in mice

Male adult C57BL/6J mice (average weight 20 g) were obtained from the Experimental Animal Center of Nanjing Medical University and maintained in a specific pathogen-free animal facility. The mice were randomly divided into three groups (*n* = 6), (I) Sham group, in which mice underwent laminectomy; (II) SCI + DMSO group, in which spinal cord contusion was conducted and equivalent DMSO solution to the SCI + ZO group injected intraperitoneally per day. (III) SCI + ZO group, in which contusion was conducted with injection of 2.5 mg/kg ZO solution. The first ZO administration was given at 1 h after SCI.

### Quantitative RT-PCR

Total RNA was extracted from cells using TRIzol after LPS treatment for 12 h. The concentration and purity of RNA were detected using UV-spectrophotometry (NanoDrop-2000, MA, USA) at 260 nm and 280 nm. Reverse transcription of RNA using a Goldenstar^TM^ RT6 cDNA Synthesis Kit (TsingKe, Beijing, China) in accordance with manufacturer’s instruction, followed by the quantification using a SYBR Green Master (TsingKe). The target genes were normalized to GAPDH using the Δ Δ ^Ct^ method. The primers were listed in Table 1.

**Table 1:** Primer sequences of quantitative reverse transcription-polymerase chain reaction.

Oligo Name	Sequence (5′ --------> 3′)	
TNF-α (Ms)	Forward	CTGAACTTCGGGGTGATCGG
	Reverse	GGCTTGTCACTCGAATTTTGAGA
IL-1β (Ms)	Forward	GCAACTGTTCCTGAACTCAACT
	Reverse	ATCTTTTGGGGTCCGTCAACT
MMP2 (Ms)	Forward	CCTGGACCCTGAAACCGTG
	Reverse	TCCCCATCATGGATTCGAGAA
MMP9 (Ms)	Forward	GCAGAGGCATACTTGTACCG
	Reverse	TGATGTTATGATGGTCCCACTTG
GAPDH (Ms)	Forward	TGACCTCAACTACATGGTCTACA
	Reverse	CTTCCCATTCTCGGCCTTG

### Western blot (WB)

Protein extraction was carried out using a Total Protein Extraction Kit (KGP2100, KenGEN) according to the manufacturer’s instructions after LPS treatment for 24 h. After concentration determination by BCA method, equivalent protein was sequentially performed electrophoresis, transferring, and blocking. Protein was then probed with primary and secondary antibodies, and measured using an enhanced chemiluminescence system (Tanon, Shanghai, China).

### ELISA

Tissues (4 mm length) were collected and homogenized on ice. Then the pro-inflammatory cytokines and chemokines from the supernatants were detected using the ELISA in accordance with the manufacturer’s protocols.

### Trans-well assay

Microglia (2 × 10^5^ cells/ well) were seeded into the upper chambers of trans-well inserts (8μm membrane pore size; Corning, Corning, NY, USA) in FBS-free medium with the respective reagents, and the lower chambers were placed into a 24-well plate filled with 500 μL complete medium containing LPS. After culturing for 24 h, the unmigrated cells on the upper membrane were cleaned using a sterile swab. The migrated cells on the lower membrane were fixed using 4% PFA for 30 min followed by staining with 0.1% crystal violet for 30 min, the images were observed under a microscope.

### Histology and immunology staining

Tissue collection and section manufacture were consistent with the previous description^48^. For histology, HE staining was performed on the sections according to the manufacturer’s protocol. For IF, sections were incubated with the primary and secondary antibodies as described previously^48^. Then sections were observed using a microscope system.

### Flow cytometry analysis (FCA)

To measure the polarization of microglia, BV2 cells in each group were collected for 30 min incubation with F4_80-PE and iNOS-FITC at 4 °C. Then cells were examined by flow cytometry (FACSVerse 8, BD Biosciences). Subsequently, the obtained data were analyzed using the FlowJo software (Version 7.6.1, Ashland, OR, USA).

### Behavioral assessment

The locomotor of mouse hindlimb was examined for 4 weeks post SCI using the Basso

Mouse Scale (BMS). Each mouse was allowed to move in an open field while two experienced researchers observed their movements and scored at 3, 7, 14, 21, 28 days. The scores of two hind limbs were averaged.

### Statistical analysis

Data from at least three independent biological experiments were exhibited as mean ± SD. Analysis more than two groups was carried out using one-way or two-way ANOVA, and analysis for two groups was done through unpaired two-tailed Student’s *t* tests. The graphs were drawn using the GraghPad Prism 6 software (San Diego, CA, USA). *P*-values < 0.05 were considered statistically significant.

## Conclusion

TAK1 inhibition attenuates neuroinflammation via blocking downstream MAPKs pathway in activated microglia. Moreover, downregulation of MAPKs pathway by blocking TAK1 activation reduced MMP2/9 expression and mitigated microglia migration, thus protecting neurological histology and function.
